# Transcranial magnetic stimulation in the management of autism spectrum disorder: Narrative review

**DOI:** 10.1192/j.eurpsy.2021.1313

**Published:** 2021-08-13

**Authors:** M. Adnan, M. Zafar, C. Trivedi, U. Beg

**Affiliations:** 1 Psychiatry, Mercy Hospital and Medical Center, Lincolnwood, United States of America; 2 Psychiatry, Central State Hospital, Milledgeville, United States of America; 3 Psychiatry, Psychiatry Austin, Austin, United States of America; 4 Psychiatry, Central State Hospital, Milledgeville, United States of America

**Keywords:** TMS, Children, Autism spectrum disorder, Repetitive Transcranial Magnetic stimulation

## Abstract

**Introduction:**

Fifty years ago, the estimated prevalence of autism was 30-60 per 10,000; now, it has increased to 18.5 per 1,000. Autism disorders are 4.3 times as prevalent among boys as among girls.

**Objectives:**

This systematic review provides an overview of the management of AD with Transcranial Magnetic Stimulation.

**Methods:**

A systematic review was conducted using (“Autism spectrum disorder” AND “Repetitive Transcranial Magnetic stimulation” AND “RTMS” OR “Children and adolescent”) in PubMed, Embase, and PsycINFO, resulted in 453 hits and finally qualified 18 studies.

**Results:**

We found 18 eligible studies, 8 randomize controlled clinical trials, 10 non-controlled clinical trials comparing TMS effects with waiting-list controls (n = 6), sham-treatment (n = 8) and no control group (n=4). There was a significant reduction of repetitive, stereotyped behaviors, irritability, social behavior, and executive function improvements with a medium-size effect. Eleven studies in this review had a moderate to high risk of bias due to small sample size, lack of blinding to treatment, and inadequate follow-up period. Four studies reported the stability of these gains in clinical outcomes for more than six months with no clarification after that.

**Conclusions:**

The data encourages the potential safety and efficacy; it provides significant evidence to support TMS’s efficacy in symptom severity reductions and improved clinical outcomes in children with autism. Therefore, future large-scale randomized controlled trials are required to conclude intervention efficacy in a larger sample size further.

